# Computational support, not primacy, distinguishes compensatory memory reorganization in epilepsy

**DOI:** 10.1093/braincomms/fcab025

**Published:** 2021-03-10

**Authors:** Joseph I Tracy, Kapil Chaudhary, Shilpi Modi, Andrew Crow, Ashith Kumar, David Weinstein, Michael R Sperling

**Affiliations:** Department of Neurology, Thomas Jefferson University, Philadelphia, PA 19107, USA

**Keywords:** memory reorganization, computational support, computational primacy, temporal lobe epilepsy, paired-associate memory

## Abstract

Temporal lobe epilepsy is associated with impairment in episodic memory. A substantial subgroup, however, is able to maintain adequate memory despite temporal lobe pathology. Missing from prior work in cognitive reorganization is a direct comparison of temporal lobe epilepsy patients with intact status with those who are memory impaired. Little is known about the regional activations, functional connectivities and/or network reconfigurations that implement changes in primary computations or support functions that drive adaptive plasticity and compensated memory. We utilized task functional MRI on 54 unilateral temporal lobe epilepsy patients and 24 matched healthy controls during the performance of a paired-associate memory task to address three questions: (i) which regions implement paired-associate memory in temporal lobe epilepsy, and do they vary as a function of good versus poor performance, (ii) is there unique functional connectivity present during memory encoding that accounts for intact status by preservation of primary memory computations or the supportive computations that allow for intact memory responses and (iii) what features during memory encoding are most distinctive: is it the magnitude and location of regional activations, or the presence of enhanced functional connections to key structures such as the hippocampus? The study revealed non-dominant hemisphere regions (right posterior temporal regions) involving both increased regional activity and increased modulatory communication with the hippocampi as most important to intact memory in left temporal lobe epilepsy compared to impaired status. The profile involved areas that are neither contralateral homologues to left hemisphere memory areas, nor regions traditionally considered computationally primary for episodic memory. None of these areas of increased activation or functional connectivity were associated with advantaged memory in healthy controls. Our emphasis on different performance levels yielded insight into two forms of cognitive reorganization: computational primacy, where left temporal lobe epilepsy showed little change relative to healthy controls, and computational support where intact left temporal lobe epilepsy patients showed adaptive abnormalities. The analyses isolated the unique regional activations and mediating functional connectivity that implements truly compensatory reorganization in left temporal lobe epilepsy. The results provided a new perspective on memory deficits by making clear that they arise not just from the knockout of a functional hub, but from the failure to instantiate a complex set of reorganization responses. Such responses provided the computational support to ensure successful memory. We demonstrated that by keeping track of performance levels, we can increase understanding of adaptive brain responses and neuroplasticity in epilepsy.

## Teaser

By distinguishing different levels of memory performance, Tracy et al. isolate the brain network characteristics uniquely associated with intact memory in epilepsy. They highlight two forms of cognitive reorganization (c*omputational primacy; computational support*) and show that intact memory requires adaptive abnormalities, and impaired memory involves failure to generate compensatory, supportive brain responses.

## Graphical Abstract

**Graphical Abstract:**
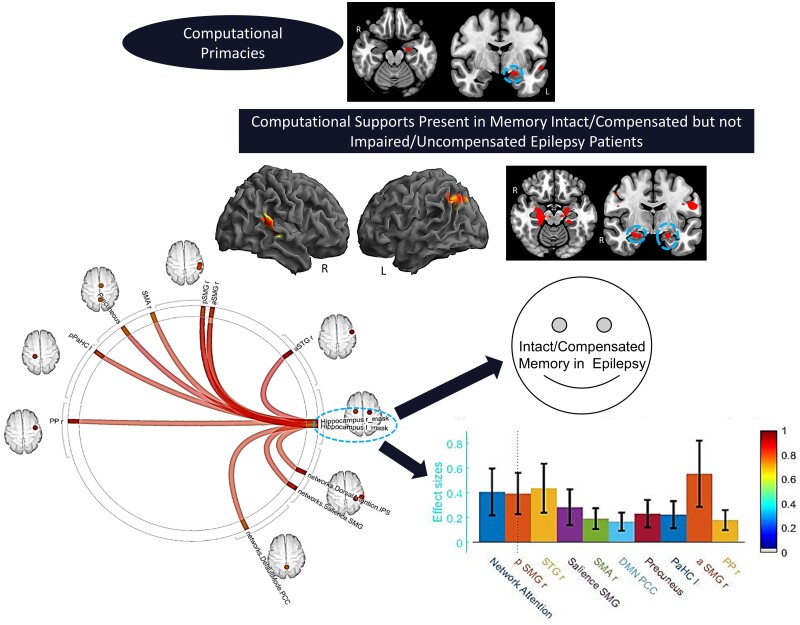

## Introduction

Temporal lobe epilepsy (TLE) is associated with cognitive impairment, most commonly in episodic memory, with up to 70% of TLE patients displaying memory problems.^1–4^ There is a substantial sub-group, however, who are able to maintain adequate episodic memory despite their temporal lobe disease.^5^ While the functional neuroanatomy of episodic memory deficits has been studied and explicated, we know very little about the regional and brain network features that support preserved memory in the setting of temporal lobe disease. The hippocampus plays a major role in seizure generation and spread in TLE,^6^ and mesial temporal sclerosis ^7^^,^^8^ involving hippocampal atrophy remains the most common pathology of focal TLE.^9^^,^^10^ The hippocampus is also critically important to the formation of episodic long-term memories, through its known specialization for associative encoding and memory consolidation emerging from animal models,^11–13^ electrophysiology ^14–19^ and task functional MRI (fMRI). For instance, task-based fMRI studies with healthy individuals have demonstrated verbal memory tasks reliably activate the medial temporal lobe including hippocampus and para-hippocampal regions, particularly when evoked by associative encoding such as paired-associate learning paradigms.^20–25^ Accordingly, impairments in learning and memory are common in TLE, and removal of the hippocampus from surgery to control seizures often has a negative effect on episodic memory.^26–31^

In the setting of temporal lobe pathology, the brain’s adaptive drive to maintain adequate memory performance can result in cognitive reorganization, involving the redistribution both of primary memory computations and alterations in the use and recruitment of the regions and networks that provide the supportive processing, i.e. the necessary but not sufficient functions to implement effective memory. Several previous studies have shown intra- and inter-hemispheric reorganization in memory encoding networks across both verbal and visual domains in individuals with left and right TLE.^32–40^ In TLE, paired-associate memory (PAM) tasks^39^^,^^41^ have been quite effective at revealing the reorganization of memory-related networks, involving structures such as the mesial temporal lobe and hippocampus. The presumptive goal of such reorganization is to compensate for the dysfunctional epileptogenic region. There has been prior work describing the general patterns of cognitive reorganization that might help maintain or restore adaptive functioning in the setting of brain disease such as TLE.^42^ What has been missing from the above studies, however, is a direct comparison of TLE patients with ‘intact’, memory functioning from those who are memory ‘impaired’. The degree to which TLE patients with intact memory actually differ from normal controls with similar levels of memory performance also remains unchartered and poorly understood. More specifically, in the setting of temporal lobe disease, little is known about the particular regional activations, functional connectivities (FCs) and/or network reconfigurations that appear distinctly prone to implement either changes in the primary computations or the supportive functions that drive adaptive plasticity and compensated memory output.^43–47^

With these advances in mind, we examined regional activation and network connectivities in TLE, but do so with a focus on important subgroups, namely, those whose memory performance suggests either ‘intact’ or ‘impaired’ brain responses. Utilizing task fMRI, we addressed three questions: First, what regions implement PAM in TLE compared to healthy controls, and do such regions vary as a function of good versus poor PAM performance? Second, are there unique FCs present during memory encoding that may account for ‘intact’ status in TLE through the preservation of primary memory computations or the supportive functions that allow for ‘intact’ performance? Third, what are the features during memory encoding that are most distinctive about ‘intact’ memory: is it the magnitude and location of regional activations during memory encoding, or the presence of enhanced functional connections to key structures such as the hippocampus?

We first tested for regional activation differences between our experimental groups, focusing on TLE group differences from healthy matched controls (HC). Next, after categorizing participants as good versus poor PAM performers, we utilized a two-factor general linear model (GLM) model to determine if such regional activations varied by Experimental Group (TLE, HC) and PAM performance, with the interaction of these factors the effect of key interest. To more carefully delineate cognitive compensation within our TLE patients, we separated TLE patients with ‘intact’ performance from those with clearly impaired PAM performance. Based upon the above performance distinctions, we then utilized generalized psychophysiological interaction techniques (gPPI) to capture the differential FC present during successfully remembered PAM trials compared to a non-memory control condition. In these gPPI analyses, our focus was on whole brain connectivity to the region(s) producing key activation differences between our performance groups, i.e. the hippocampus, a region with well-established importance to episodic memory functioning. Lastly, we leveraged the classification power of support vector machine (SVM) Learning, to identify the features in our data that best discriminated ‘intact’ versus ‘impaired’ memory performance in TLE. More specifically, SVM determined whether the magnitude of regional effects sizes on the PAM task, as opposed to the FCs mediating successful encoding, constituted better classifiers of ‘intact’ memory status. The goal of this last analysis was to isolate the characteristics most robustly associated with truly successful verbal memory encoding and adaptive brain functional reorganization in TLE.

## Materials and methods

A total of 54 patients with drug-resistant unilateral TLE (left = 32; age ranges: 21–62; right = 22; age ranges: 24–64) matched on age, handedness^48^ and gender were recruited from the Thomas Jefferson University Comprehensive Epilepsy Centre. All patients were determined to be surgical candidates for either a standard anterior temporal lobectomy or thermal ablation of the ictal mesial temporal lobe based upon a multimodal evaluation including neurologic history and examination, scalp video-EEG, MRI, PET and neuropsychological testing.^49^ All participants were left hemisphere language dominant, as verified by an fMRI verb generation task. All patients had a Full-Scale IQ of 80 or higher (WAIS-IV, The Psychological Corporation).^50^ Additional details on study entry criteria, including the age, gender-matched healthy controls (HCs, *n* = 24; age ranges: 23–57) are provided in Supplementary Section 1. The demographic and clinical characteristics of the experimental groups are presented in Table 1. Definitions for the terms computational support, compensatory primacy, paired-associate memory and SVM learning are described in Box 1.

### Paired-associate memory task

To capture both deleterious memory effects and the potential neuroplastic responses to maintain effective episodic encoding and recall, we chose to trigger memory relevant BOLD activation through a PAM paradigm, based upon items and modifications of the Wechsler Memory Scale.^51^ The PAM task design comprised three phases: encoding, when the word pairs are learned and memorized; distraction, when a set of intervening arithmetic problems are solved; cued recall, when one member of the target pair is presented and the participant must speak aloud the second member of the pair. The task design is described in Supplementary Figs 1 and 2.

The word pairs that were successfully recovered during the cued recall period were considered successfully encoded. The cued recall responses of forgotten and successfully encoded pairs for each subject were used to calculate the subsequent forgetting (SFE) and subsequent memory (SME) effects. SMEs involved PAM encoding trials subsequently remembered, with SME trials analysed only for those participants with eight or more trials remembered (at least 32% recall rate). The activations associated with these SME trials minus the math control condition constituted the main contrast of interest. SFE trials involved encoding trials not subsequently remembered, with the SFE minus math contrast analysed only for those participants with 8 (32%) or more forgotten trials. Participants with an SME of 60% or better (proportion correctly recalled, at least 16 of 25 pairs correct) were classified as Good PAM performers; participants with lower SME scores were classified as Poor PAM performers. The Cohen’s *d* effect sizes for these subgroups were huge [HC (Good versus Poor), *d* = 2.69; left temporal lobe epilepsy (LTLE) (Good versus Poor), *d* = 3.41; right temporal lobe epilepsy (RTLE) (Good versus Poor), *d* = 2.82], using the criteria for *d* reported by Sawilowsky^52^ [(0.01–0.19) = very small, *d* (0.02–0.49) = small, *d* (0.5–0.79) = medium, *d* (0.8–1.19) = large, *d* (1.2–1.99) = very large, *d* (≥2.0) = huge]. Within the TLE group, we developed a separate grouping strategy to identify performances at the more extremes of the accuracy continuum, considering those with an SME of 70% or better to have displayed fully ‘intact’ recall, and those with an SME of 30% or lower to have displayed ‘impaired’ recall performance (n.b., Cohen’s *d* effect size was huge (*d* = 6.55) for this subgroup). These accuracy levels and performance groupings were based on well-established and widely used Neuropsychological Norms.^53^ Note, the HC and RTLE patients generally performed better on the PAM task, with few individuals below 70% in terms of recall accuracy (see Table 1), creating too large an imbalance to make Experimental Group comparisons possible using this ‘intact’ versus ‘impaired’ distinction.

**Table 1:** Sample demographic, clinical, PAM task and neuropsychological data

		HC	LTLE	RTLE	*P*
No.		24	32	22	-
Age, year. (*M* ± SD)		38 (9.1)	43.5 (14.5)	40.9 (12.6)	0.27
Education, year (*M* ± SD)		16.6 (2.6)	15.6 (2.4)	14.2 (2.2)	0.22
Gender, M:F		15:9	19:13	10:12	0.19
Edinburgh Handedness R/L		22/2	29/3	21/1	-
Age at Epilepsy onset (*M* ± SD; years)		NA	23 (18.2)	19.6 (13.14)	0.1
Duration of Epilepsy (*M* ± SD; years)		NA	21.3 (19)	20.8 (16.8)	0.12
Seizure focality (with/without GS or 2nd GS)		NA	14/18	10/12	0.5
Temporal pathology (NB/HS/T/D/E/ASI)		NA	6/15/2/1/4/4	10/8/1/0/1/2	0.24
HS/Non-HS			15/17	8/14	0.21
Seizure type:					
CPS		NA	6	5	
CPS/SPS		NA	4	2	
CPS + 2nd GS		NA	5	3	
CPS/SPS + 2nd GS		NA	7	5	
CPS+GS		NA	8	4	
CPS/SPS+GS		NA	2	3	
fMRI PAM task data					
PAM task accuracy (M±SD)		16 (6.02)	12.7 (5.8)	14.04 (5.4)	0.13
PAM performance group: Good/Poor		14/10	14/18	10/12	
PAM performance group: Intact/Impaired		11/2	7/12	12/3	
Neuropsychological data					
Full-Scale IQ (WAIS-IV)		NA	92.1 (12.5)	94.3 (12.7)	0.12
CVLT-II total learning (M±SD)		NA	42.3 (11.4)	48.5 (5.2)	0.01 ^a^
Epilepsy medication		NA			
VGNC	CBZ, OXC, LTG, PHT	NA	18	12	
GABAa agonist	PB, BZD, Pr	NA	4	3	
SV2a receptor mediated	LVA	NA	4	4	
CRMP2 receptor mediated	LCM	NA	3	2	
Multi-action	VPA, TPM, ZNS	NA	3	1	
VGCC	PGB, GBP	NA	2		

Table depicts the mean (standard deviation) for the demographic information and neuropsychological test data, with *P*-values (except where indicated) derived from independent sample *t*-tests (two-level experimental group factor) and one-way ANOVAs (three experimental group factor).

Temporal pathology was confirmed by neuroradiologists during presurgical MRI scans: NB = normal brain; HS = hippocampal sclerosis; T = tumour; D = dysplasia; ASI = abnormal signal intensity; seizure type: SPS = simple partial seizure; CPS = complex partial seizure; 2nd GS = secondary generalized tonic-clonic seizure; GS = generalized tonic-clonic seizure; M = male; F = female; CVLT = California Verbal Learning Test; WASI = Wechsler Abbreviated Scale of Intelligence; PAM = paired-associate memory Task; WAIS = Wechsler Adult Intelligence Scale, General ability index, Verbal comprehension index; WMS = Wechsler Memory Scale, Logical memory, Auditory memory, Verbal Paired Associate; VGNC = Voltage-gated sodium channel blockers: CBZ = carbamazepine; OXC = oxcarbazepine; PHY = phenytoin, GABAa Agonist: Gamma aminobutyric acid a receptor agonist: PB = barbiturates; BZDs = benzodiazepines (diazepam, lorazepam, clonazepam, clobazam); SV2a receptor-mediated AEDs: LVA = levetiracetam; CRMP2 receptor-mediated AEDs: LCM = lacosamide; VGCC = voltage-gated calcium channel: PGB = pregabalin; GBP = gabapentin; Multi-action AEDs: VPA = valproate; TPM = topiramate; Pr = primidone.

a Statistically significant;

**Table 2:** MNI coordinates of significant cluster maxima for subsequent memory effect minus math contrast demonstrating performance group differences within HC and LTLE

Region	Peak MNI coordinates			
	K	X	y	Z
(A) HC (Good minus Poor)				
R Anterior/superior frontal gyrus	108	14	60	16
L Anterior/superior frontal gyrus	78	−6	56	16
R Anterior/middle temporal gyrus	83	40	4	−32
L Superior parietal gyrus	34	−24	−68	58
R Frontal pole	26	22	58	−2
(B) LTLE (Good minus Poor)				
L Supramarginal gyrus	248	−62	−52	20
L Superior temporal gyrus	177	−44	−24	6
L Precuneus	81	−16	−56	34
L Superior parietal lobule	60	−32	−54	38
R Angular Gyrus	46	38	−56	28
L Hippocampus	43	−36	−22	−8

Subpeaks of the interest are also included. The cluster thresholds correspond to corrected false discovery rRate with significance level of height *P *<* *0.05.

**Table 3:** MNI coordinates of significant cluster maxima for subsequent memory effect minus math contrast from ANOVA interaction effect and performance group *t*-test within LTLE

Region	Peak MNI coordinates			
	k	x	y	z
(A) Two-Way ANOVA Interaction, Factor 1 (LTLE, HC) and Factor 2 (Good, Poor)				
L Superior parietal lobule	1016	−16	−52	34
L Fusiform gyrus	523	−38	−74	−8
L Posterior/superior temporal gyrus	358	−46	−26	6
R Thalamus	322	10	−34	8
L Central operculum	292	−56	−4	4
L Heschl’s Gyrus	226	−46	−26	6
L Hippocampus	103	−28	−24	−16
(B) *T*-test, LTLE PAM Groups (Intact minus impaired)				
R Posterior superior temporal gyrus	609	58	−58	14
R-Heschl’s Gyrus	377	38	−28	6
L Supramarginal/angular gyrus	215	−40	−70	40
R Cerebellum	177	16	−62	10
L Hippocampus	75	−20	−24	−18
R Hippocampus	72	14	−22	14

Activated cluster description for Interaction; (A) LTLE and HC groups, (B) LTLE (Intact versus Impaired) group. Subpeaks of the interest are also included. The cluster thresholds correspond to corrected false discovery rate with significance level of height threshold (*P *<* *0.05).

**Table 4:** Multivariate classification analysis (SVM) within LTLE utilizing significant activation effect sizes and gPPI FC coefficients (with Seed) as input

Model: Response Variable: Intact minus Impaired Status Based on PAM Task					
Region (Effect Sizes)	CA (%)	AUC	SE	*P*-value	95% CI
RHCP	54	0.633	0.154	0.406	0.330–0.935
RSTG	78.2	0.898	0.085	0.013^*^	0.732–1.0
LHCP	76.1	0.878	0.096	0.018^*^	0.689–1.0
LAG	81.2	0.959	0.050	0.004^*^	0.861–1.0
RHG	79	0.939	0.067	0.006^*^	0.807–1.0
RCBLM	56	0.512	0.119	0.910	0.278–0.746
Region (FC Coef.)					
aSTGr	54.2	0.551	0.167	0.749	0.224–0.878
aSMGr	74.3	0.796	0.123	0.045^*^	0.554–1.0
pSMGr	53	0.510	0.164	0.949	0.188–0.832
SMAr	52.2	0.510	0.164	0.949	0.188–0.832
Precuneus	51	0.490	0.167	0.949	0.163–0.816
pPaHCl	49.8	0.408	0.168	0.565	0.078–0.738
PPr	78.2	0.918	0.075	0.009^*^	0.772–1.0
DMN.PCC	46.2	0.388	0.159	0.482	0.077–0.699
Networks.Salience.SMG	46	0.327	0.155	0.277	0.022–0.631
Networks.Attention.IPS	54	0.531	0.168	0.848	0.201–0.860

* Statistically significant; CA = classification accuracy; AUC = area under curve; SE = standard error; CI = confidence interval.

**Box 1:** Definitions

(1) C*omputational primacy:* Specific brain component processes necessary for successful cognitive output. In the case of episodic memory recall this may involve regional (and inter-regional) brain activity and communications that produce successful associative encoding and consolidating of elements into a stored memory engram.
(2) *Computational support*: Brain cognitive operations, outside of primary computations, that support and enhance but are not absolutely necessary for successful cognitive output. In the case of episodic memory recall this may involve brain activity or communications in the regions or networks responsible for the dedication of attentional resources, implementation of cognitive control strategies, engagement of task-relevant stimuli, the conduct of working memory, the generation of lexical associations, the enhancement of memory search functions, the conduct of working memory, initiate the readiness of input processes such as audition.
(3) *PAM task:* A verbal learning and recall task composed of the following phases: encoding of single word pairs, calculation of intervening arithmetic problems and the visual presentation of a previously viewed single word as a cue for recall of the other member of the original target pair.
(4) *SVM learning:* A classification technique that finds the hyperplane in N-dimensional space that distinctly classifies data points, utilizing, in addition, methods such as cross-validation. In the setting of this study, the data points are the regional activations and network connections that best distinguished intact from impaired memory performances in the TLE patients.

### MRI data acquisition and fMRI preprocessing pipeline

The fMRI scan was obtained on a 3T Philips Achieva MRI scanner for all participants using an eight-channel head coil. Participants were instructed on all phases of the PAM task and response requirements before entering the scanner. FMRI data preprocessing for PAM task was performed on each subject using fMRIPrep 1.5.8^54^ based on Nipype tool 1.4.1.^55^ Details of the MRI acquisition parameters, and fMRIprep pipeline are provided in Supplementary Section 3.

### General linear model fMRI analyses

Utilizing SPM12,^56^ one or two sample *t*-tests were carried out to determine whole brain significant activations emerging from the contrast of interest (SME minus math control), either within or between experimental (LTLE, RTLE, HC) or performance groups (Good minus Poor; Intact minus Impaired) (*P*_FDR_ corrected <0.05, cluster level). We also analysed the activation associated with the SFE (SFE minus math condition contrast) and tested for group differences in whole brain activation. A two-factor ANOVA analysing the above SME contrast utilized Experimental Group (e.g. LTLE, HC) and PAM performance (Good versus Poor) as independent variables, with their interaction the effect of interest (*P*_FDR_ corrected <0.05, cluster level).

### Generalized psychophysiological interaction analyses

We performed gPPI analysis to verify the key PAM task modulated connectivity effects (SME effect versus math control condition) utilizing the CONN toolbox v17.f.^57^ Whole brain gPPI was applied with the seed based upon the results of the key GLM analyses noted above (see Supplementary Section 4 for gPPI details). The first gPPI model was based on the results of the significant interaction effect from the two-factor ANOVA on the SME contrast [Experimental Group (LTLE versus HC) and PAM performance (Good, Poor)]. Our second gPPI model was based upon the significant *t*-test result comparing the Intact and Impaired LTLE groups on the SME activation. These task modulation-dependent gPPI measures allowed us to determine the strength of FC to the seed(s) with the whole brain as the search set (*P*_FDR_ corrected <0.05, two tailed).

### Support vector machine models and analyses

A linear SVM algorithm (MATLAB, R2018a, with cross-validation through the leave one out method) was used for multivariate classification analysis of Intact versus Impaired memory performance in LTLE. We used as inputs the magnitude of the significant regional activations on the PAM task given by the key ANOVA differences emerging from the Intact/Impaired comparison within LTLE, as well as the FCs associated with a key regional SME effect distinguishing Intact/Impaired status (hippocampi bilaterally). This SVM was done solely within the left TLE group as too few patients in the RTLE group displayed impaired performance. To assess the classification success of the SVM model, we computed receiver operating characteristic curves, with associated area under the curve data (AUC, *P* < 0.05). The goal of the SVM model was to select the features from our GLM task activation and gPPI FC data that best discriminated successful verbal memory.

Linear SVM regressions on PAM accuracy as a response (continuous variable) was also carried out within the LTLE group (Intact and Impaired performers; and separately for LTLE Good and Poor performers), with the relevant, significant PAM activation effect sizes and gPPI FC coefficients as predictors (5-fold cross-validation).

### Statistical analysis of demographic, clinical and behavioural data

Statistical analyses were conducted using (IBM^®^ SPSS^®^ v24), with alpha level set at *P* < 0.05 for multiple comparisons. *T*-tests or chi-square tests were used, as appropriate, to determine differences in our experimental groups on demographic/clinical characteristics, IQ and a neuropsychological memory measure available on the TLE patients (Table 1). Also, a one-way ANOVA was utilized to determine if the three Experimental Groups (HCs, LTLE and RTLE) differed on PAM task accuracy.

### Data availability

The data that support the findings of this study are available within the article and its Supplementary material. Additional data relevant to the study can be provided upon request to the corresponding author.

## Results

### Clinical, demographical and behavioural results

Our sample clinical, demographical and behavioural data can be seen in Table 1. Our three experimental groups not differ in age, gender or educational background. Left handedness was identified in a small number of participants as indexed by the Edinburgh Handedness scale.^48^ Age at epilepsy onset, duration of epilepsy illness and the rate of mesial temporal sclerosis did not differ in the LTLE and RTLE groups (Table 1). As expected, there was significantly worse performance in the left compared to right TLE group on a neuropsychological measure of verbal learning and encoding, the CVLT-II Total Learning [t(df = 52) = −2.4, *P* < 0.01].

### Experimental group differences in PAM task activation

We examined the pattern of brain activation associated with the SME contrast within each of our Experimental Groups (Supplementary Fig. 3). In HCs, the significant clusters of activation were predominantly in the dominant left hemisphere involving the left inferior frontal, left hippocampal/parahippocampal gyri, left parietal/occipital and left lingual regions. The RTLE group also showed a predominantly left hemisphere pattern, with left hippocampal, left inferior frontal and left inferior parietal/occipital activations. In the LTLE group, there was bilateral activation in the hippocampus, middle temporal, inferior frontal and inferior parietal/occipital regions. The LTLE group showed clear and strong evidence of right hemisphere involvement (middle temporal and inferior frontal), with such activity absent in the other groups. All the above activated regions were statistically significant.

**Figure 1:**
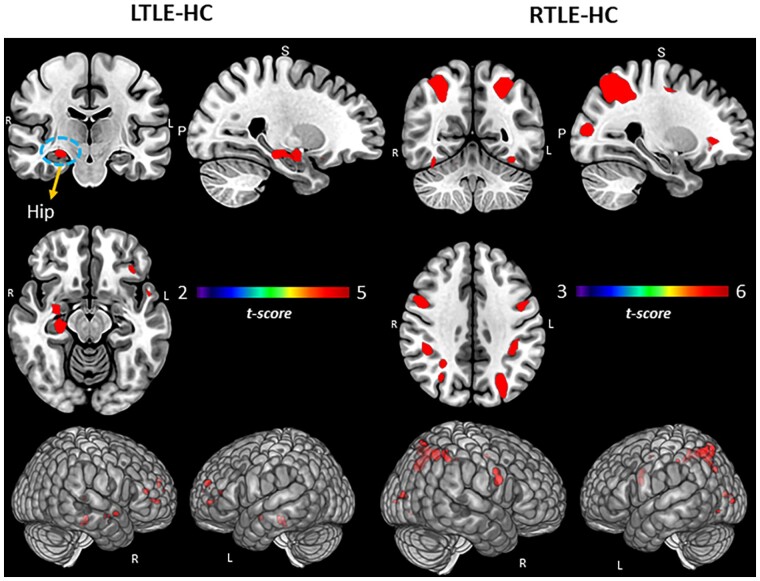
**Group comparisons for TLE and HCs.** Regional activation associated with SME minus math condition contrast. Surface rendering and slices in vertical panels show significant activation for LTLE versus HC and RTLE versus HC groups. *P*_FDR_ corrected *t*-statistic <0.05, cluster level. Hip = hippocampus.

In terms of experimental group differences involving the SME (Fig. 1; Supplementary Table 1), compared to HCs, the LTLE group showed increased activation in the right hippocampus/parahippocampus, along with increased activity in the bilateral middle/superior frontal region. In contrast, compared to HCs, the RTLE group showed a limited set of differences, with the areas of increased activation involving bilateral posterior/superior regions, parietal cortex, and the occipital lobe.

We also examined whether the experimental groups differed with regard to the SFE minus math contrast. Neither the LTLE nor the RTLE groups differed from HCs in this regard, suggesting that in contrast to the group differences that emerged for successful memory encoding, the functional neuroanatomy associated with forgetting is similar in our TLE groups relative to controls.

### PAM task performance group activation differences by GLM

**Figure 2:**
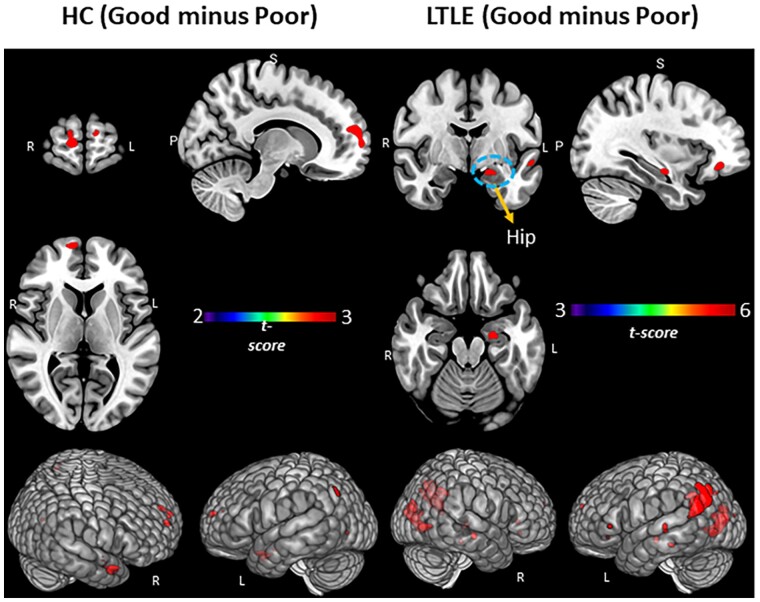
**PAM performance (Good versus Poor) within the LTLE and HCs.** Regional activation associated with SME minus math condition contrast. Surface rendering and slices in vertical panels show activation for Good minus Poor subgroups of HC and LTLE. *P*_FDR_ corrected *t*-statistic <0.05, cluster level. Hip = hippocampus.

We examined the unique patterns of SME-related activations associated with Good versus Poor performance within each of our Experimental Groups (see Table 2A and B, Fig. 2). In the HCs, compared to the Poor performers, the Good PAM Performers showed activations in bilateral anterior/superior frontal, left superior parietal, as well as right anterior/middle temporal lobe. In LTLE, compared to the Poor PAM performers, the Good performers showed several left-sided activation increases (hippocampal, supramarginal, and superior temporal, superior parietal). Within RTLE, no reliable activation differences were present between the Good and Poor performance groups.

**Figure 3:**
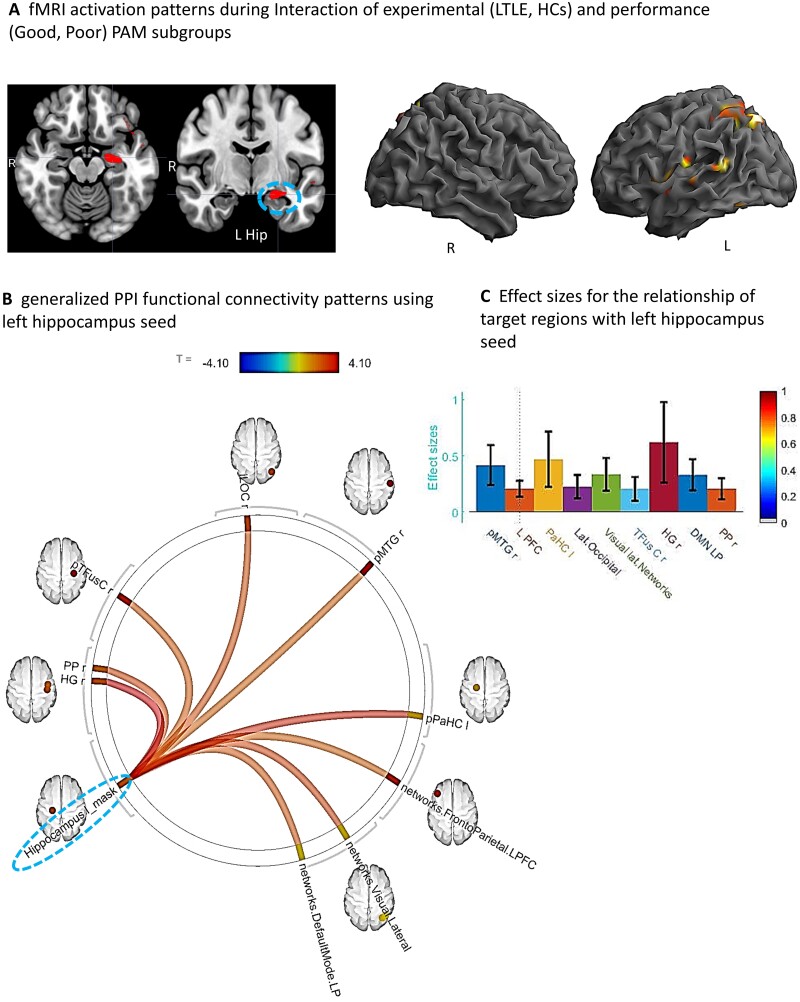
**Interaction of experimental (LTLE, HCs) and performance (Good, Poor) groups.** (**A**) Whole brain surface rendering and slices revealing significant activation resulting from interaction in two-way ANOVA on SME minus Math contrast. Factor One, Experimental Group (LTLE, HCs); Factor 2, PAM performance (Good, Poor; t-statistic *P*_FDR_ corrected < 0.05, cluster level). Results highlight the areas where the SME/Math difference is significantly larger in LTLE patients compared to HCs on the PAM task. (**B**) FC results of gPPI using left hippocampal region emerging from the GLM interaction effect (see panel A) as seed. Seed cluster (MNI coordinates of maxima: −28 –24 −16; 103 voxels), involving the time course of the SME minus Math contrast was extracted and the generated gPPI regressor effects (gPPI_β) are displayed. Results revealed nine target regions with functional relationship of statistically significant strength with left hippocampal seed (*P*_FDR_*t*-statistic corrected < 0.05). (**C**) Bar diagram depicts effect size (mean beta weights from gPPI) for each functional relationship between the source seed (left hippocampus) and target region.

We next sought to determine if the regional activation differences associated with Good versus Poor PAM performances varied as a function of TLE or HC status. We utilized a two-factor ANOVA model, with the interaction between these factors the effect of interest. The results showed that the Good versus Poor SME activation differences did vary as a function of LTLE or HC group (see Table 3A and Fig. 3A). More specifically, in LTLE compared to HCs, the Good PAM performers displayed significantly greater activation than the Poor performers in the left hippocampus, and several left hemisphere regions (superior parietal, fusiform, posterior superior temporal, central operculum and Heschl’s gyrus). The interaction effect involving PAM performance (Good versus Poor) and the RTLE and HC groups produced no statistically reliable results.

**Figure 4:**
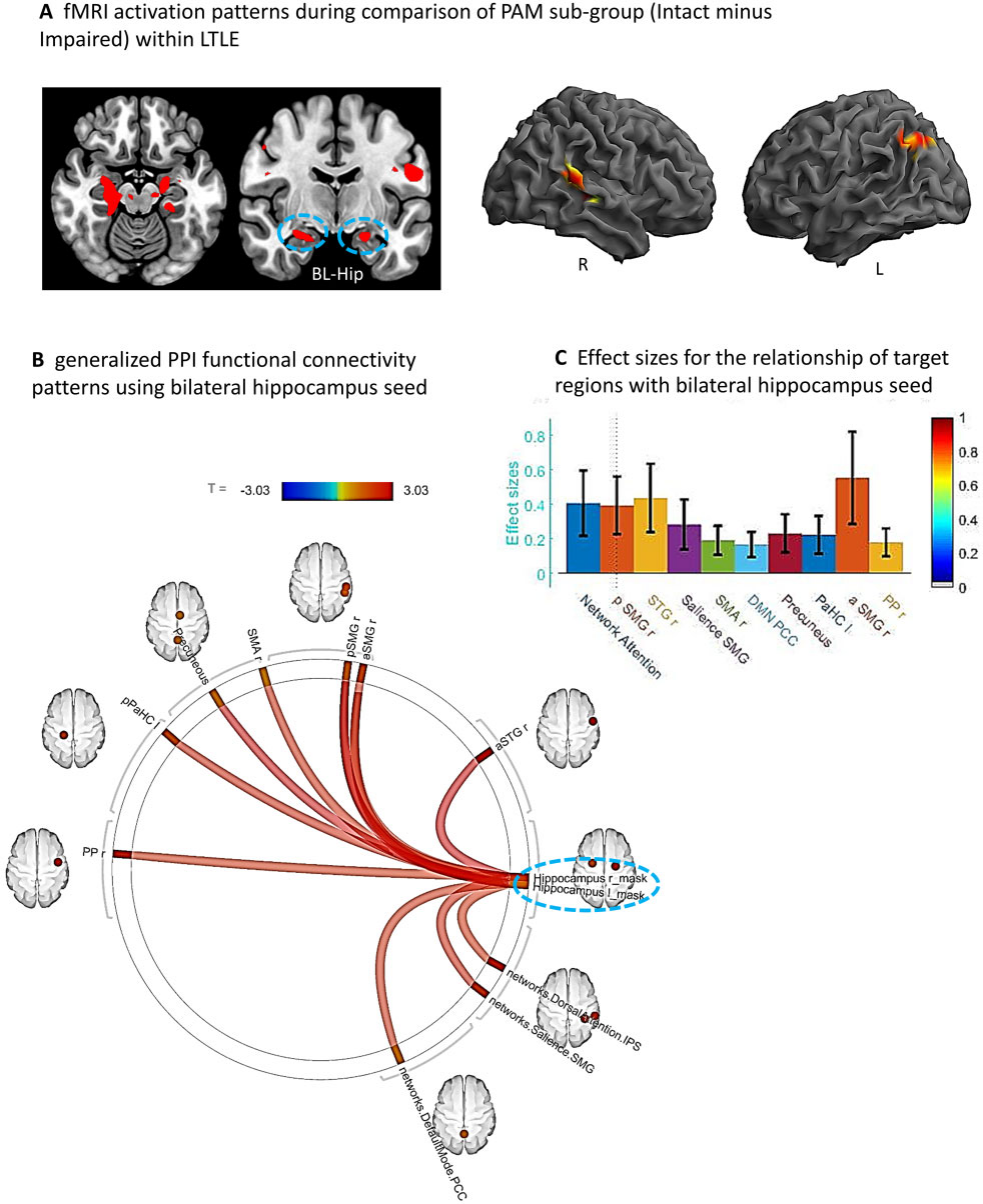
**Sub-group comparison (Intact minus Impaired) within LTLE.** (**A**) Whole brain surface rendering and slices revealing significant activation (SME minus Math contrast) resulting from *t*-test of sub-group difference (intact minus impaired) within the LTLE patients (*P*_FDR_*t*-statistic corrected, <0.05, cluster level). Results highlight the areas where the SME/Math difference is significantly larger in the LTLE patients with intact compared to impaired performance levels on the PAM task. (**B**) FC results of gPPI using bilateral hippocampal region emerging from the *t*-test effect (see **A**) as seed. Seed clusters of left (MNI coordinates of maxima: −20 –24 −18; 75 voxels) and right (14 –22 14; 72 voxels) hippocampus, involving the time course of the SME minus Math contrast was extracted and the generated gPPI regressor effects (gPPI_β) are displayed. Results revealed 10 target regions with functional relationship of statistically significant strength with combined hippocampal seed (*P*_FDR_*t*-statistic corrected < 0.05). (**C**) Bar diagram presented effect size (mean beta weights from gPPI) for each functional relationship between the source seed (combined left and right hippocampus) and target regions.

To delineate cognitive compensation in our TLE patients, we examined the SME effects associated with the more extreme ends of PAM performance within our TLE groups. These results showed that in LTLE, clearly Intact compared to Impaired memory performance was associated with increased activation in both the left and the right hemispheres (see Table 3B; also Fig. 4A). Importantly, we observed not only bilateral hippocampal activation, but also right hemisphere activation (posterior superior temporal, Heschl’s gyrus, cerebellum), and left angular gyrus.

### Functional connectivity effects (gPPI) with key GLM results as seed

Having established that activation in the left hippocampus is a key region distinguishing Good and Poor performances in LTLE compared to controls (see Table 3A), and that activation in the bilateral hippocampus is important to the Intact and Impaired distinction within LTLE (Table 3B), we next sought to determine if there are FCs to these key areas that serve as mediators of successful memory during the PAM task (i.e. mediate SME trials but not the math control condition). To accomplish this, we utilized gPPI, focusing on the whole brain connectivity related to the hippocampal seed given by the above GLM analyses.

With regard to the Good versus Poor difference in LTLE and HC, we found strong FC between nine regions and the left hippocampal seed during the SME trials, as presented in Fig. 3B and C. These regional FCs involved mostly right-sided regions (planum polar, Heschl’s gyrus, temporal fusiform, lateral occipital, posterior middle temporal gyrus). Other regions that showed increased connectivity to the left hippocampus were the left parahippocampus and parts of well-established intrinsic FC networks such as the default mode (right lateral parietal), visual lateral (right side) and fronto-parietal (left prefrontal cortex) networks. Of these regions, the right Heschl’s, right posterior middle temporal and left parahippocampal regions showed the strongest FC with the left hippocampus.

With regard to the Intact versus Impaired difference within LTLE, using the combined bilateral hippocampi seed, we found 10 regions that showed strong FC during the SME trials (see Fig. 4B and C). These regional FCs involved mostly right hemisphere regions (planum polar, anterior/posterior supramarginal, superior temporal and supplementary motor area), in addition to the left parahippocampus. Other regions connected to the bilateral hippocampi were members of well-established intrinsic FC networks such as the dorsal attention (right inferior parietal sulcus), salience (right supramarginal gyrus) and default mode (precuneus, posterior cingulate cortex) networks. The right anterior/posterior supramarginal gyrus, right superior temporal gyrus and dorsal attention network region (right inferior parietal sulcus) showed the strongest FC with the bilateral hippocampi.

To examine the issue of laterality for the case where bilateral hippocampal activity was found during the PAM task (i.e. found in the *t*-test identifying the task activation unique to the Intact group in LTLE), we re-ran the gPPI once for the left and then separately for the right hippocampal seed. The results showed that the network communicating with the left hippocampal seed in the Intact LTLE group involved left parahippocampus, supplementary motor area, anterior and posterior right supramarginal gyrus, dorsal attention network region (right inferior parietal sulcus) and default mode (precuneus, posterior cingulate cortex). In contrast, the network communicating with the right hippocampal seed in the Intact LTLE group involved right planum polar, right anterior superior temporal gyrus and salience network region (right supramarginal gyrus). The regions emerging from these separate hippocampal seeds yielded results that together are identical to the combined hippocampal seed.

### SVM prediction of Intact versus Impaired status in LTLE

To further understand the features in our data that best discriminated intact from impaired performance in LTLE, we utilized SVM multivariate classification analyses focused on determining whether SME-related activation magnitudes (see Table 4, effect size variables) or the strength of FCs with the bilateral hippocampi seed used in our gPPI model (see Table 4, FC coefficient variables) were better classifiers. For the PAM task, six variables produced statistically significant (*P* < 0.05) AUC values of 0.796 or higher, mostly involving right hemisphere variables. Four of these six variables involved SME activation effects (right hemispheric superior temporal, Heschl’s gyrus; left hemispheric hippocampus, angular gyrus), with two FC variables reaching statistical significance as a classifier (right anterior supramarginal and right planum polar gyri).

Lastly, we examined whether the above subset of significant variables remained reliable and effective classifiers after including FSIQ or neuropsychological memory (CVLT-II Total Learning) in the model and re-running the identical SVM. The results showed that the above six variables remained significant in the SVM. FSIQ was not a significant predictor (AUC, *P*-value = 0.48), however, the neuropsychological measure did emerge as significant (AUC, *P*-value < 0.05), suggesting that our Intact/Impaired grouping was consistent with the baseline memory levels of our LTLE patients. We examined in LTLE the correlation between our SVM predictor set (16 variables) and age at epilepsy onset and duration of epilepsy. Duration bore no significant relations, but age of epilepsy onset was negatively correlated with two variables, the left precuneus (*r* = −0.56, *P* < 0.05) and right planum polar area (*r* = −0.59, *P* < 0.05). These findings indicated that earlier epilepsy onset was associated with stronger FC modulation between those two regions and the bilateral hippocampi seed. Age of onset was not significantly different [*t*(df, 17) = −0.99, *P* = 0.33] between the Intact/Impaired groups [Intact, onset = 14.7 (12.93) years; Impaired, onset = 23.1 (19.76) years]. Nonetheless, we ran the identical SVM and found the same six variables remained significant in terms of classifying our Intact/Impaired LTLE patients, with age of onset also emerging as a significant classifier (AUC = 0.84, *P* < 0.05).

SVM regression analyses were carried out (5-fold cross-validation) utilizing PAM accuracy (continuous variable) as a response, with the predictors involving the significant activation effect sizes and gPPI FC coefficients utilized in the primary SVM reported above classifying Intact versus Impaired LTLE performers (see Table 4). This SVM regression model showed good fit to the observed data [RMSE = 0.21, *R*^2^ = 0.64], replicating the primary model, and, most importantly, explained a good proportion of the variance in the classification response. This verified that the same set of 16 predictors of Intact/Impaired status also predicted PAM accuracy as a continuous measure. To further test whether the main findings reported in the SVM (Table 4) also distinguished Good versus Poor performance in LTLE, we ran a linear regression to test if the significant activation effect sizes (regions shown in Table 3a for the interaction of Good/Poor and Experimental Group) and gPPI FC coefficients (regions displayed in Fig. 3) predicted PAM accuracy. This SVM regression model was able to discriminate the Good and Poor groups in LTLE with roughly the same level of fit to the data as the Intact/Impaired grouping LTLE [RMSE = 0.17, *R*^2^ = 0.23], but substantially less variance was explained, suggesting the model captured less effectively and meaningfully the features that most distinguish the groups in a manner that would have generalizability. A difference in the SVM models was expected as the two grouping methods reflect quantitatively different levels of memory success. This difference may suggest that the regional activations and FCs implementing successful memory are dynamic and vary as performance levels change. These analyses make the point that the variables we report in our main SVM model (Table 4) capture the features unique to the extreme and high end of performance, with such features less characteristic of individuals with lower memory performance levels.

## Discussion

We utilized both activation magnitude and FC to demonstrate the regional mechanisms involved in the reorganization of episodic memory in temporal lobe compromised epilepsy patients. We carefully linked our fMRI analyses to successfully encoded words through the use of SME effects. SME contrasts reflecting activation relative to a math control condition did show reliable regional effects within each of our experimental groups, but an investigation of unsuccessful PAM performance (SFE) showed no reliable effects in any of our groups (HC, LTLE or RTLE). The latter makes clear that merging these distinct cognitive performances (trials) likely hides the unique computational properties related to compensatory, successful memory reorganization. Our data showed that left, but not right, TLE differed from healthy controls in their brain maps of PAM SMEs, with the maps of the LTLE patients more distinctly right-sided, particularly in mesial temporal areas, as well as showing bilateral superior frontal activation compared to controls.

The above findings, however, did not take into account performance levels. Indeed, most unique to our study is the examination of different memory performance levels, captured by categorizing our participants as Good versus Poor PAM performers, and, in the case of our TLE patients, as clearly Intact versus Impaired. Several distinctive features of Good performance in LTLE relative to HC were present. For instance, there was increased activation in left mesial (left hippocampal) and several left posterior regions (superior parietal and temporal, fusiform, central operculum and Heschl’s gyrus; see Table 3A, Fig. 3A). These left hemisphere areas seemed to be uniquely and abnormally upregulated among the Good LTLE performers. We found no reliable Good versus Poor activation differences within RTLE, or when comparing RTLE to HCs.

An added layer of our investigation of performance effects involved use of a stringent criterion, one that separated performance levels so as to be sure to isolate truly intact versus impaired recall levels relative to same age peers (SME at 70% accuracy or better; 30% or worse). This distinction showed that, indeed, a different set of regions were responsible for these higher, truly compensated performance levels. Intact as compared to Impaired performance was associated with bilateral hippocampi, left supramarginal/angular gyrus, right posterior superior temporal gyrus, and other right-sided areas (Heschl’s, cerebellar) of activation. These data made clear that in contrast to Good performance levels, Intact or heightened performance levels were strongly right hemisphere in nature, involving uniquely increased activity in the (language) non-dominant hemisphere contralateral to the ictal focus.

These more stringent performance-based changes were unique to LTLE. For instance, while the RTLE group did show some PAM activation differences relative to HCs in bilateral parietal areas (see Fig. 1; Supplementary Section Table 1), both RTLE and HCs showed a similar strong left hemisphere pattern, involving the left hippocampus and left inferior frontal regions (see Supplementary Fig. 3). On the PAM task, the HCs brain regional involvement did also vary with performance. Good, advantaged memory performance was characterized by bilateral activity involving the anterior/superior frontal lobe, left superior parietal, and right anterior/middle temporal regions (Table 2, Fig. 2). A conjunction analysis of Good performers in the HC and LTLE groups showed a common left-sided activation pattern (hippocampus, inferior frontal, middle temporal, superior temporal, and left angular gyrus, lingual gyrus), indicating that some of the ipsilateral effects observed in LTLE are, indeed, normative and not unusual brain responses. An area notable because of its very limited presence in the normative responses of the Good and Intact LTLE groups involved frontal cortex, as the frontal lobe was a prominent part of the Good performance response in HCs.

Our data also made clear that most of these shared (i.e. normative) areas of activation differed from and, therefore, were not critical to Intact/Compensated, or even Good performance in LTLE. In brief, the advantaged areas in the HCs were more anterior/middle temporal and frontal in location compared to the advantaged LTLE performers. With the above in mind, we can see that in terms of activation magnitude the areas of increased activity in the clearly intact/compensated LTLE patients that are non-normative involved the right hippocampus response, the right posterior superior temporal gyrus, and the other right hemisphere clusters, responses clearly missing in the other groups. Note, the less accurate, but Good, memory performances in LTLE that were non-normative involved left superior parietal, left fusiform, left central operculum, left Heschl’s gyrus and the right thalamus. With the exception of the right hippocampus, which has been observed in normals during paired-associate paradigms,^20^^,^^21^ the non-normative areas we found to be unique to the LTLE Good and Intact performers are not considered primary computational regions for episodic memory (i.e. implementing associative encoding, memory consolidation). This unique profile of non-normative areas suggested these represent forms of computational support, as opposed to areas of computational primacy, for the production of successful episodic memory. Accordingly, our data provided strong evidence that when seeking to clarify the regions most unique to implementing successful, truly compensated memory reorganization in LTLE, it is important to distinguish between performance levels.

To more specifically characterize the brain features associated with intact/compensated status in LTLE, we looked not just at PAM activation magnitude, but also changes in FC unique to successful PAM encoding. The goal of these gPPI analyses was to find the areas communicating during encoding with the key regions present in our activation magnitude data, taking advantage of our knowledge that such regions (hippocampi) play a critical role in primary memory computations based upon a literature dating back decades.^13^^,^^58^^,^^59^ Any observed significant FCs can be said to reflect regions with activity synchronized and correlated with the hippocampus seed, implying that they mediate successful memory, even though their activation magnitude may not have been sufficient to appear in our GLM findings. The results revealed that nine regions had reliable FC with the left hippocampus in Good relative to the Poor group in LTLE compared to HCs (see Fig. 3A). These regional FCs implicate extensive SME-related communication between the left hippocampi and right-sided regions, in addition to contact with regions that are part of intrinsic networks known either to be associated with memory processes (default mode), or other processes often associated with a strong memory response to viewed material (executive function, fronto-parietal network; visual lateral network). These results stand in contrast to the activation magnitude data which showed that the Good/Poor groups in the LTLE and HCs differed predominantly in left-sided areas.

In our attempt to understand the communication circuitry underlying truly intact/compensated status in LTLE, our gPPI model (bilateral hippocampal seed) revealed 10 regions with reliable FC during encoding. Consistent with the right hemisphere nature of the PAM activation magnitude data, we saw mostly right-sided FCs associated with Intact status. Perhaps not unexpectedly, the bilateral hippocampi showed connectivity to regions that are part of intrinsic functional networks well-associated with either memory encoding processes (e.g. the default mode network, precuneus, posterior cingulate; see our work^60^), or other cognitive computations that can be seen as important for a successful memory (e.g. dorsal attention and salience networks). These FCs gave us a window through which to view the broader networks supporting compensated memory in these temporal lobe compromised patients. As a test of whether these FCs were in any sense normative, we conducted the identical gPPI model in HCs (bilateral hippocampal seed). The resulting gPPI was not significant (*P*_FDR_ corrected <0.20), with very different mediating regions in the model. This follow-up analysis demonstrated that the 10 regions mediating memory performance in the Intact group did not reflect normal increases in FC during the SME trials.

Our performance focused gPPIs were similar in that both implied strong communication with the non-ictal, non-dominant right temporal areas in the advantaged LTLE groups, as well as strong connections to the same intrinsic connectivity network, the Default Mode Network (DMN), known for its association with episodic memory. Both gPPIs also showed connectivity to the left, ipsilateral parahippocampus. The two gPPIs, however, differed with respect to the other intrinsic networks recruited to support successful memory (the fronto-parietal network for distinguishing Good versus Poor LTLE status; the dorsal attention and salience networks for the separation of Intact/Impaired status). The literature does suggest that these three intrinsic networks bear some functional similarity. For instance, each of these networks involve top-down responses that are phasic forms of cognitive control, each is triggered by exogenous stimuli, and each evokes a cognitive goal that establishes what stimuli are important, with subsequent initiation and maintenance of task focus and engagement.^61^ This complex, but shared functionality, can be viewed as set of facilitative communications that support episodic memory. It is important to be reminded that many of the regions forming these memory-related connectivities with the hippocampus were not activation hotspots in the linked PAM activation data.

Lastly, having established that distinct performance levels produced different task-related activations and memory-mediated FCs, we sought to determine which feature(s) best distinguished Intact versus Impaired status in LTLE, noting that this grouping provided the most compelling contrast to argue that our findings truly represented compensatory memory reorganization. Utilizing AUC values from the SVM model as our guide (see Table 4), we found that activation magnitudes were generally better classifiers of Intact/Impaired status on the PAM, with these regions involving a mix of right and left hemisphere structures. The reliable FC predictors involved right hemisphere-based connections (right anterior supramarginal gyrus, right temporal planum polar) to the bilateral hippocampal seeds. Looking across these predictors, it is really the pattern involving right posterior temporal (magnitudes: superior temporal gyrus, Heschl’s; FCs: temporal planum polar) and nearby right inferior parietal features (FC: supramarginal gyrus) that was most striking. Thus, our SVM results clearly demonstrated the importance of right hemisphere structures in compensatory memory responses in LTLE, making clear that with the exception of the left hippocampus, the critical predictors of Intact/Compensated status were not homologues of the left hemisphere areas known to associate with primary episodic memory computations in normals.

A potential implication of our results is that truly compensatory memory reorganization in LTLE can be understood to take two forms. One form involves change to the regions conducting *primary memory computations* such as associative learning, memory engram formation and consolidation. Relevant to this, our SVM data indicated that no fundamental reorganization occurred with regard to *computational primacies*, as left hippocampal SME-related activation remained one of the best predictors of Intact/Compensated status. The second form of reorganization involves change to the regions providing *computational support* to the core and primary memory processes. These support regions reflect recruitment of cognitive functions that facilitate, and perhaps even are a necessity, for strong, highly successful memory performance. Relevant to this, our SVM data indicated that there were non-normative regional involvements mostly in the right temporal lobe providing *computational support* for memory processing either through task-relevant upregulations in activity or task-mediated increases in connectivity to the bilateral hippocampi. One might speculate as to the cognitive nature of these supportive functionalities (e.g. dedication of attentional resources, cognitive control/strategy, engagement of task-relevant stimuli, working memory, increased reliance on lexical association or search functions, auditory processing), but the fact that none of these areas discriminated Good versus Poor performance in HCs suggested the regions identified by our SVM model were unique to the implementation of successful memory in LTLE. Our PAM activation magnitude and FC data pointed to other regional differences between the Intact/Impaired groups, providing additional clues to the functionalities that may have provided computational support for strong, successful episodic memory in LTLE (see Tracy and Osipowicz^42^ for discussion of alterations in cognitive networks that might drive adaptive performance in the face of disease). Ultimately, however, our data remained silent on the specific role played by the regions we have identified as strongly associated with compensated memory organization in LTLE.

Several methodologic considerations are pertinent to mention. Follow-up examination revealed that our SVM model is capturing activation magnitude and FC effects that cannot be accounted for by cognitively relevant baseline characteristics of our LTLE sample (IQ, neuropsychological memory), or clinical characteristics such as hippocampal volume (see Supplementary Section 6), anti-epileptic medication, epilepsy duration, or age or illness onset. Interestingly, our data do point to the possibility that earlier onset for LTLE lays down a set of increased connectivities not just to the ictal but also the non-ictal hippocampus. Other methodologic and conceptual considerations are noted in Supplementary Section 7. These involve the likely importance of white matter changes to cognitive reorganization, and the potential relevance of task-mediated communication changes with other regions, not just the hippocampi. Also, in describing forms of memory reorganization, while we noted several hemispheric patterns, we emphasize that such reorganization processes must be understood at the functional/regional, not hemispheric level. Lastly, we acknowledge that we did not capture individual patterns of reorganization, and the forms of compensatory reorganization we describe for LTLE may not hold for all cognitive processes/tasks, nor for all forms of epilepsy.

Our data showed that the brain reorganizations implementing Intact/Compensated versus Impaired/Uncompensated memory performance in LTLE reflects a complex substrate. The theme throughout is that non-ictal, non-dominant posterior temporal regions are most important, recruiting both increased regional activity (posterior superior temporal, Heschl’s gyrus) and increased modulatory communication with the hippocampi, all features that are missing in the Impaired/Uncompensated LTLE patients. The right hemisphere areas that emerged as most important are neither contralateral homologues to left hemisphere areas, nor are they areas traditionally considered computationally primary for episodic memory. Yet, the story is complex as activation increases in ictal-side areas also appear to be necessary recruitments. Importantly, none of these areas of increased activation, nor FCs, were associated with advantaged (Good) episodic memory in healthy controls, making clear their unique association with compensatory memory status in LTLE.

Our emphasis on different performance levels yielded insight not just into the regional changes in activation magnitude and FC that are most crucial to compensated episodic memory, but makes clear that in doing so different forms of cognitive reorganization emerge and should be distinguished. Namely, regions can reflect a change in *computational primacy*, and in this respect, our Intact/Compensated LTLE group showed little change relative to HCs as the hippocampi and left parahippocampus, as well as regions that are part of an intrinsic network linked to memory (the default mode), remained important distinguishing features. Our performance effects also revealed a set of regions that likely provided *computational support*, with our data demonstrating that in this respect our Intact/Compensated LTLE did show differences, i.e. adaptive abnormalities, relative to HCs, involving mostly the right posterior temporal lobe, as well as regions that are members of intrinsic networks that could readily provide functionalities to enhance memory through increased communication with the bilateral hippocampi.

Our data provide evidence of changes in computational support, as opposed to computational primacies, not seen in healthy controls and missing in performance disadvantaged LTLE groups. In so doing, we isolated unique regional activations and mediating FCs that implement truly compensatory reorganization in LTLE. Our results provide a new perspective of memory deficits in TLE, as it is not just a knockout of a key functional hub such as the hippocampus that causes deficits. Deficits may also arise from a failure to instantiate a complex set of reorganization responses capable of preserving memory. Such responses, whether they involve increases in regional activation or increases in FC with core memory structures, provide the computational support to ensure effective memory performance.

### Clinical application

Despite the extensive research focusing on the mesial temporal lobe and episodic memory functioning in disorders such as TLE,^6^ we know very little about the regional and brain network features that support preserved memory in the setting of temporal lobe disease. In order to succeed, advances in treatments aimed at fostering recovery from memory dysfunction will need to precisely know these brain features, and the reorganization principles governing them. By keeping track of performance levels and different types of cognitive reorganization responses, our study increases understanding of adaptive brain responses and the neuroplasticity of episodic memory. As personalized treatment strategies such as brain stimulation, precision surgery or targeted drug delivery are increasingly used to ameliorate neurologic pathology such as epilepsy, we believe our novel results reveal a set of relevant features that can be a valuable guide to help patients preserve or obtain intact and compensated memory status.

## Supplementary Material

fcab025_Supplementary_Data

## Abbreviations

AUC =: area under curve
FC =: functional connectivity
fMRI =: functional MRI
gPPI =: generalized psychophysiological interaction
HC =: healthy control
LTLE =: left temporal lobe epilepsy
PAM: paired-associate memory task
RTLE =: right temporal lobe epilepsy
SVM =: support vector machine
SME =: subsequent memory effect
SFE =: subsequent forgetting effect
TLE: = temporal lobe epilepsy
